# Rethinking biomarker strategy in gastric cancer immunotherapy: from tumor to host

**DOI:** 10.3389/fimmu.2026.1847526

**Published:** 2026-06-11

**Authors:** Nabil Ismaili

**Affiliations:** 1 Department of Medical Oncology, Mohammed VI Faculty of Medicine, Mohammed VI University of Sciences and Health (UM6SS), Casablanca, Morocco; 2 Mohammed VI Foundation of Sciences and Health (FM6SS), Rabat, Morocco; 3 Oncopathology, Biology and Environment of Cancer Laboratory, Mohammed VI Center of Research and Innovation (CM6RI), Rabat, Morocco

**Keywords:** autoantibodies, biomarkers, ctDNA, gastric cancer, gut microbiome, host-tumor integration, immunotherapy, MSI

## Abstract

Immune checkpoint inhibitors (ICIs) have transformed advanced gastric cancer (GC) treatment, but durable responses remain rare, highlighting the need for better patient selection. Recent studies suggest that host-derived autoantibodies (e.g., ANA, ENA) may serve as prognostic markers in GC patients receiving immunotherapy. These hypothesis-generating observations indicate that pre-existing humoral immunity could reflect a clinically relevant axis of immune fitness. This review critically appraises these findings alongside established and emerging predictive biomarkers. We examine the strengths and limitations of PD-L1, MSI, TMB, and EBV status, and explore the clinical potential of dynamic tools like ctDNA and computational models. We also discuss emerging evidence on intrinsic resistance to PD-1 blockade in MSI-H GC, including PTEN mutations, low TMB within MSI-H tumors, and antigen presentation defects. Murine models have provided key insights into these resistance mechanisms and the immunomodulatory role of the gut microbiome. Collectively, the data support a shift from single-analyte biomarkers toward integrative, dynamic, systems-level models for patient selection, heralding a new era of precision immune-oncology in GC. However, most emerging biomarkers remain investigational and require prospective validation.

## Graphical Abstract

**Figure:**
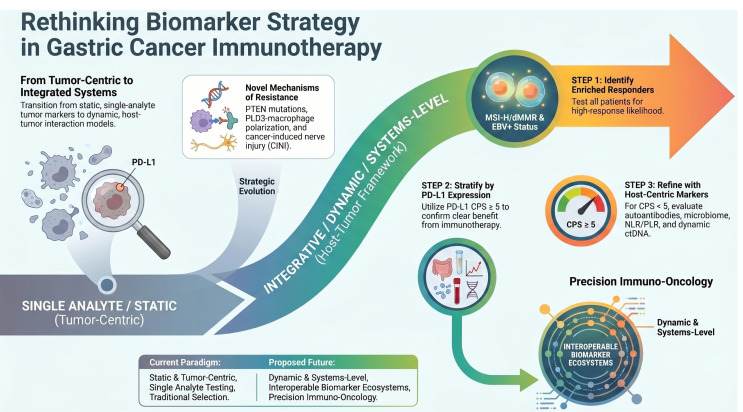

## 1 Introduction

Gastric cancer (GC) remains a major global health problem, ranking as the fifth most common malignancy and fifth leading cause of cancer-related death worldwide, with an estimated 970,000 new cases and 660,000 deaths in 2022 (1).

The advent of immune checkpoint inhibitors (ICIs) has been a major therapeutic advance, improving overall survival (OS) in phase III trials such as CheckMate 649, KEYNOTE 859, and ORIENT 16, particularly in programmed death ligand one (PD-L1)-enriched populations [2−4]. More recently, this success has extended to the perioperative setting: the MATTERHORN trial showed that adding durvalumab to FLOT chemotherapy significantly improved event-free survival (EFS), with OS data still maturing (2).

Despite these advances, only a subset of patients derive durable benefit from ICIs, while others face toxicity and high costs without gain. This heterogeneity underscores the urgent need for reliable predictive biomarkers. Current clinical decisions rely on PD-L1 expression and microsatellite instability (MSI) status, but these are imperfect: many PD-L1-negative or microsatellite stable (MSS) patients respond, while some biomarker-selected patients do not (3, 4).

The molecular heterogeneity of GC has been comprehensively characterized by The Cancer Genome Atlas (TCGA), which identified four subtypes: EBV-positive, MSI, chromosomal instability (CIN), and genomically stable (GS) (5). This classification has profound implications for immunotherapy response: EBV-positive and MSI tumors show the highest immune infiltration and PD-L1 expression, whereas CIN and GS tumors are less immunogenic (6, 7).

Recent studies have explored a new dimension by investigating the prognostic value of host-derived autoantibodies in GC patients receiving immunotherapy (8). These works provide a compelling rationale for looking beyond the tumor to the patient’s systemic immune milieu. The humoral immune response against tumor-associated antigens (TAAs) is well documented across multiple cancer types, with autoantibodies detectable months to years before clinical diagnosis (9).

This review critically appraises these emerging findings within the broader biomarker landscape. We examine canonical biomarkers, discuss the promise and perils of emerging technologies (circulating tumor DNA [ctDNA], machine learning), and propose a conceptual framework integrating host-centric and tumor-centric biomarkers. Notably, except for MSI/deficient mismatch repair (dMMR) and PD-L1 Combined Positive Score (CPS), most biomarkers discussed are exploratory and not yet validated for routine clinical use.

## 2 Canonical biomarkers: strengths, limitations, and unresolved questions

### 2.1 PD-L1 expression

Despite its widespread use, PD-L1 remains a clinically pragmatic yet biologically imprecise biomarker, reflecting an adaptive immune-resistance state that can fluctuate over time and across tumor sites (4). Limitations include spatial and temporal heterogeneity, variable scoring systems, and different antibody clones and cut-offs (3). PD-L1 is evaluated using the tumor proportion score (TPS) or the CPS, with CPS increasingly preferred in GC because of its stronger association with ICI benefit in pivotal trials. In CheckMate 649, benefit was greatest with CPS ≥ 5, but many regulatory approvals are not strictly restricted by PD-L1 expression, acknowledging that some PD-L1-negative patients also benefit (4, 10). Importantly, a large meta-analysis by Lu et al. showed that multiplex immunohistochemistry/immunofluorescence (mIHC/IF) had significantly higher diagnostic accuracy (AUC = 0.79) than PD-L1 IHC alone (AUC = 0.65) across multiple tumor types (11).

### 2.2 Microsatellite instability and Epstein-Barr virus status

These are the most robust predictive biomarkers (12–14). MSI high (MSI-H) tumors, driven by deficient mismatch repair, harbor thousands of mutations, leading to a high neoantigen load and profound immune infiltration. MSI-H accounts for 5-10% of advanced GCs (15-20% of early stage). Similarly, EBV-positive GCs (approximately 8-10% of all GCs) exhibit a strong immune signature with high PD-L1 expression (14). Both subtypes show high response rates to ICIs (frequently >50% in MSI-H), although evidence for EBV positivity derives largely from retrospective analyses and small cohorts (15).

However, not all MSI-H GCs respond equally to PD-1 blockade. In a phase II trial of pembrolizumab in MSI-H GC, Kwon et al. reported an objective response rates (ORR) of 55.6% but identified significant heterogeneity (16). Patients with higher TMB within the MSI-H subgroup had superior outcomes (median TMB 37 vs. 23 mutations/Mb in responders vs. non-responders; P = 0.0018). Furthermore, PTEN mutations were associated with poor response. These findings underscore the need for additional molecular stratification even within the MSI-H subtype.

### 2.3 Tumor mutational burden

The tumor-agnostic approval of pembrolizumab for TMB-high (≥10 mutations/Mb) solid tumors does not necessarily imply disease-specific predictive validity in GC, where MSI-H accounts for many TMB-high cases. This threshold has not been prospectively validated in GC. CheckMate 649 did not find TMB to be predictive independent of MSI status (10, 17), possibly because of the dominant influence of MSI-H tumors and the relative scarcity of TMB-high, MSS tumors in GC. However, emerging data suggest TMB retains predictive value even within MSI-H GC, with higher TMB linked to deeper responses and longer progression-free survival (PFS) (16, 18). Prospective validation in GC-specific cohorts is required before TMB can be routinely used independent of MSI status (17).

### 2.4 PTEN mutations and PI3K/AKT/mTOR pathway

Accumulating evidence implicates phosphatase and tensin homolog (PTEN) loss-of-function mutations as a negative predictor of ICI response in GC. In a study of 45 MSI-H/dMMR gastrointestinal tumors treated with PD-1 blockade, Chida et al. found that PTEN mutations (especially in the phosphatase domain) were significantly associated with lower ORR (12.5% vs. 54.8%; P = 0.049) and shorter PFS (2.6 vs. 15.6 months; HR 5.04; P < 0.001) (18). Tumors with PTEN phosphatase domain mutations exhibited an immunosuppressive microenvironment (decreased intratumoral cluster of differentiation 8+ [CD8+] T cells, increased CD204+ tumor-associated macrophages, and PI3K (Phosphoinositide 3-kinase)/AKT (Protein Kinase B)/mTOR (mammalian target of rapamycin) pathway enrichment). These data suggest that PTEN mutations confer intrinsic resistance to PD1 blockade and may identify patients who would benefit from PI3K/AKT/mTOR targeted combinations (18, 19). However, these findings require validation in larger prospective studies (19).

### 2.5 Beyond PD-1: Other immune checkpoint molecules as potential biomarkers

While the PD-1/PD-L1 axis dominates clinical practice, other immune checkpoints have emerged as potential biomarkers and therapeutic targets. Lymphocyte activation gene 3 (LAG-3) is an inhibitory receptor on exhausted T cells; its co-expression with PD-1 identifies a more dysfunctional T cell subset. High LAG-3 expression on tumor-infiltrating lymphocytes in GC has been associated with poor prognosis and may predict resistance to anti-PD1 monotherapy (20). T cell immunoreceptor with Ig and ITIM domains (TIGIT) is another inhibitory receptor on natural killer (NK) cells and T cells (including tumor-infiltrating regulatory T cells [Tregs]). TIGIT expression correlates with PD-1 expression, and dual blockade shows synergistic antitumor effects in preclinical models. T cell immunoglobulin and mucin domain-containing 3 (TIM-3) is upregulated on exhausted CD8+ T cells and implicated in anti-PD1 resistance. Its expression on intra-tumoral CD8+ T cells in GC correlates with advanced stage and poor prognosis. The spatial distribution and co-expression patterns of these molecules within the tumor microenvironment (TME) may serve as more sophisticated biomarkers than any single marker alone (21, 22).

### 2.6 Inflammatory markers: neutrophil-to-lymphocyte ratio and beyond

Neutrophil to lymphocyte ratio (NLR) is a simple, inexpensive prognostic biomarker in GC patients receiving ICIs. Gou et al. (2021) showed that pretreatment elevated NLR (cut off 3.23) was significantly associated with inferior PFS (3.9 vs. 7.9 months; HR 0.42, P < 0.001) and OS (6.3 vs. 13.5 months; HR 0.38, P < 0.001) in metastatic GC patients receiving anti-PD1 agents, with multivariate analysis confirming NLR as an independent prognostic factor (23). Similarly, Zurlo et al. (2022) found that in locally advanced GC receiving neoadjuvant chemotherapy, low NLR (<2.5) was associated with better PFS, OS, and pathological response (24). A meta-analysis by Tan et al. (2024) confirmed that high NLR and platelet to lymphocyte ratio (PLR) are associated with poorer OS in ICI-treated GC patients, with median NLR cut-offs ranging from 2.5 to 5.0 across studies (25). The discordance highlights the need for standardized cut-offs and prospective validation. Nonetheless, these markers remain attractive due to low cost and universal availability, and may still hold value as part of a composite score (26).

Beyond NLR, other peripheral blood ratios have been investigated. The Treg-to-effector T cell ratio reflects the balance between immunosuppressive and cytotoxic immune populations; a high intratumoral Treg/CD8 ratio has been associated with poor prognosis and resistance to immunotherapy. The myeloid-derived suppressor cell (MDSC) to-neutrophil ratio may provide additional discriminatory power, as MDSCs are potent immunosuppressors that expand in GC. Innate lymphoid cell 1 (ILC1)-to-ILC3 ratios represent emerging innate lymphoid cell biomarkers; ILC1s promote T helper Cell 1 (Th1) responses while ILC3s are associated with inflammation and immunosuppression. However, these ratios require flow cytometry and are not yet standardized for routine clinical use. Additionally, Bai et al. (2021) reported that lower absolute monocyte count, lower platelet-to-lymphocyte ratio (PLR), and lower lactate dehydrogenase (LDH) were independently associated with improved OS in patients receiving ICIs across multiple tumor types, further supporting the utility of peripheral blood parameters (27). Standardization of cut-offs and prospective validation are required before these markers can be integrated into clinical algorithms.

### 2.7 CD4+ and CD8+ T cell exhaustion status as biomarkers

The functional status of CD4+ and CD8+ T cells can be assessed by flow cytometry and may serve as predictive biomarkers. Exhausted CD8+ T cells are characterized by co-expression of multiple inhibitory receptors (PD1, TIM-3, LAG-3) and diminished effector function (impaired Interferon-gamma [IFN-γ], Tumor Necrosis Factor alpha [TNF-α], and granzyme B production). In GC, high frequencies of PD-1+TIM-3+ exhausted CD8+ T cells in pretreatment peripheral blood correlate with poor response to anti-PD1 therapy. Conversely, the presence of stem-like progenitor exhausted CD8+ T cells (T-cell factor 1 [TCF-1]+PD1+) has been associated with better ICI outcomes, as these cells retain proliferative capacity and can differentiate into effector cells upon checkpoint blockade. Multiparametric flow cytometry panels can simultaneously assess exhaustion markers, proliferative capacity (Ki-67), and cytokine production to generate composite exhaustion scores (28, 29).

CD4+ T cell subsets also carry prognostic significance. T follicular helper (Tfh) cells support B cell responses and tertiary lymphoid structure formation, which have been linked to favorable ICI responses. In contrast, Tregs (CD4+CD25+FOXP3+) suppress antitumor immunity. The ratio of effector CD4+ T cells (Th1, Th17) to Tregs may predict ICI responsiveness. Furthermore, NK cell functionality (assessed by CD107a degranulation or expression of activating receptors like NKG2D, NKp30) represents an additional biomarker dimension, as NK cells contribute to innate antitumor immunity and can be reinvigorated by ICIs. The critical cells expressing PD-1, CTLA-4, LAG-3, and TIGIT include not only T cells but also NK cells, B cells, and myeloid populations, necessitating comprehensive immunophenotyping (30, 31).

### 2.8 Anti-CTLA-4 antibodies and Treg depletion: controversies and evidence

The mechanism of action of anti-CTLA-4 antibodies remains controversial, specifically whether therapeutic efficacy depends on Treg depletion versus CTLA4 blockade on effector T cells. Preclinical studies have shown that anti-CTLA4 antibodies with Fc domains capable of mediating antibody-dependent cellular cytotoxicity (ADCC) deplete intratumoral Tregs, contributing to antitumor efficacy. Simpson et al. (2013) demonstrated in mouse models that IgG2a anti-CTLA4 antibodies, but not IgG1 variants, depleted Forkhead box P3-positive (FoxP3+) Tregs within the tumor microenvironment via Fcγ receptor engagement, leading to enhanced CD8+ T cell responses and tumor regression (32). Selby et al. (2013) similarly reported that anti-CTLA4 antibodies of the IgG2a isotype enhanced antitumor activity through intratumoral Treg reduction (33).

However, the situation in humans is less clear. Sharma et al. (2019) found that ipilimumab treatment did not deplete FOXP3+ Tregs in tumor tissues from melanoma, prostate, and bladder cancer patients, suggesting that other mechanisms may predominate (34). Arce Vargas et al. (2018) reported that Fc effector function contributes to the activity of human anti-CTLA4 antibodies, with the FCGR3A (CD16) genotype influencing clinical outcomes (35). A contrasting hypothesis posits that anti-CTLA4 antibodies primarily block CTLA4-mediated inhibition on CD8+ T cells during the priming phase in lymph nodes, rather than depleting Tregs (36).

Furthermore, CD28 stimulation has been suggested to diminish Treg frequencies, as CD28 co-stimulation is critical for Treg development and maintenance. However, studies have produced conflicting results: while CD28 superagonists expand Tregs in some contexts, CD28 blockade may reduce Treg numbers. In the context of CTLA4 blockade, CD28 signaling is enhanced because CTLA4 normally outcompetes CD28 for CD80/CD86 binding. This enhanced CD28 signaling could potentially expand Tregs, counteracting any direct Treg depletion. The net effect likely depends on the specific antibody isotype, Fc receptor engagement, and the TME context. Therefore, interpretation of anti-CTLA4 mechanisms requires careful consideration of these conflicting data, and future trials should incorporate comprehensive immunophenotyping to resolve these controversies (37, 38).

### 2.9 Helicobacter pylori infection

A novel and intriguing biomarker is the status of *H. pylori* infection. Che et al. conducted a retrospective study of 77 patients with advanced GC treated with anti-PD-1 antibodies and found that *H. pylori* infection was significantly associated with worse outcomes. The disease control rate was 72.1% in *H. pylori*-negative versus 47.1% in *H. pylori*-positive patients (P = 0.027). Median OS was 17.5 vs. 6.2 months (HR 2.85; P = 0.021), and median PFS was 8.4 vs. 2.7 months (HR 3.11; P = 0.008). Multivariate analysis confirmed *H. pylori* infection as an independent predictor of PFS (HR 1.90; P = 0.022). The mechanism may involve *H. pylori*-induced alterations in the gut microbiome and immunosuppressive effects on dendritic cells, leading to impaired CD8+ T cell responses. These findings suggest that *H. pylori* status could serve as a readily available biomarker for stratifying GC patients likely to benefit from ICI therapy, but these observations are hypothesis-generating and should be confirmed in prospective studies (39).

### 2.10 The gut microbiome and microbial metabolites as immunomodulators

Emerging evidence implicates the gut microbiome as a critical modulator of ICI response. Zhang et al. (2026) demonstrated in a phase I trial that fecal microbiota transplantation (FMT) from healthy donors combined with anti-PD-1 therapy was safe and feasible in patients with ICI-refractory microsatellite-stable GC, with an ORR of 25% (40). Responders exhibited successful colonization of donor-derived immunogenic microbes, particularly short-chain fatty acid (SCFA) producing bacteria, and an activated peripheral immune profile.

Gao et al. (2026) comprehensively reviewed gastric microbiota-mediated immune remodeling in GC (41). Key mechanisms include: (1) bacterial metabolites such as butyrate, enhancing CD8+ T cell function via G-protein-coupled receptor 109A (GPR109A) activation; (2) Fusobacterium nucleatum recruiting tumor-associated neutrophils (TANs) expressing PD-L1 via the interleukin 17/nuclear factor kappa-light-chain-enhancer of activated B cells/v-Rel Avian Reticuloendotheliosis Viral Oncogene Homolog B (IL-17/NF-κB/RelB) pathway; and (3) Bifidobacterium enhancing dendritic cell maturation and type I interferon signaling, thereby improving ICI efficacy.

Liao et al. (2025) recently identified succinate as a key microbial metabolite that drives immunosuppressive macrophage polarization. Succinate accumulation in the TME, derived from both tumor cells and gut microbiota, activates SUCNR1 (GPR91) signaling on tumor-associated macrophages, promoting M2-like polarization and IL-10 production while suppressing CD8+ T cell function. This succinate-mediated pathway represents a potential therapeutic target to enhance ICI efficacy (42). Additionally, other metabolites such as indole-3-acetic acid, urolithin A, and spermidine have been shown to promote antitumor immunity through Aryl hydrocarbon Receptor (AhR) activation, mitophagy enhancement, and autophagy induction, respectively.

The gut microbiome composition also predicts ICI response across cancers. Higher abundance of Akkermansia muciniphila, Ruminococcaceae, and Bifidobacterium species has been consistently associated with favorable responses to PD-1 blockade. Microbiome-derived biomarkers may therefore complement existing tumor-centric markers, though standardization and prospective validation are urgently needed (43, 44). **Table 1** summarizes the main predictive biomarkers for immunotherapy in gastric cancer, including their clinical utility, limitations, and current validation status.

**Table 1:** Key predictive biomarkers for immunotherapy in gastric cancer. .

Biomarker/Ref	Type	Clinical utility	Key limitations	Current clinical validation status
PD-L1 (CPS) (6, 16, 101)	Tissue IHC	Stratifies benefit from ICI + chemo in 1st-line (esp. CPS ≥5)	Heterogeneity, variable cut-offs, temporally unstable	Phase III-validated, clinically implemented
MSI/dMMR (16, 18)	Tissue PCR/IHC	Identifies “super-responders”; high response rate to ICI monotherapy	Low prevalence (~5-10% advanced; 15-20% early); heterogeneity within MSI-H	Phase III-validated, guideline-endorsed
EBV status (5, 7, 15)	Tissue ISH/IHC	Identifies immune-rich tumors with high response rates	Low prevalence (~5-8%); retrospective data only; no dedicated phase III trials	Strong phase II/retrospective data; not guideline-mandated
TMB (16, 17, 52)	NGS (tissue/blood)	Proxy for neoantigen load; debated predictive value independent of MSI	Not standardized in GC; threshold (≥10 mut/Mb) not prospectively validated	Tumor-agnostic approval; not GC-specific validated
PTEN mutations (18, 19)	NGS (tissue)	Loss-of-function mutations (esp. phosphatase domain) associated with poor ICI response	Low prevalence (~10-15% in GC); predictive value strongest in MSI-H tumors	Retrospective; exploratory
*Helicobacter pylori* infection (39)	Serology, UBT, stool antigen	Negative infection status associated with better ICI outcomes	Conflicting data; mechanism unclear; requires prospective validation	Retrospective; exploratory
Autoantibodies (ANA/ENA) (8, 98, 99)	Serum (IIF/CLIA)	Reflects host immune activation; associated with better PFS in retrospective studies	Heterogeneity of patterns; prognostic vs. predictive unclear	Retrospective; exploratory
NLR/PLR (23, 25)	Serum (CBC)	Simple, inexpensive indicator of systemic inflammation	Cut-off not standardized (NLR range 2.5-5.0); conflicting data	Meta-analytic; non-prospective
Treg/effector T cell ratio (34, 36, 37)	Flow cytometry (blood/tissue)	High ratio predicts poor ICI response and worse survival	Requires fresh samples; no standardized cut-off	Exploratory; needs prospective validation
CD8+ T cell exhaustion score (28, 29)	Flow cytometry/scRNA-seq	High PD-1+TIM-3+ exhausted CD8+ T cells predict poor ICI response	Complex multiparametric readout; no standardized scoring	Exploratory
NK cell functionality (30, 31)	Flow cytometry (CD107a, NKG2D)	Low degranulation/activating receptor expression predicts ICI resistance	Requires fresh blood; not standardized	Exploratory
Gut microbiome composition (40–44)	Metagenomic sequencing (stool)	Enrichment of *A. muciniphila*, *Bifidobacterium*, SCFA producers associated with ICI response	High inter-individual variability; no consensus signature	Retrospective/prospective cohorts; not clinically implemented
Microbial metabolites (succinate, butyrate, indole derivatives) (42)	Metabolomics (stool, plasma)	Succinate promotes M2-like TAMs; butyrate enhances CD8+ T cell function	Complex metabolomics; causality not fully established	Preclinical/translational; exploratory
PLD3-high macrophages (82)	Tissue IHC/RNA	High PLD3 expression in TAMs suppresses TLR9 and CD8+ T cell activation	Validated only in murine models; human data lacking	Preclinical; exploratory
Neuronal injury markers (ATF3, IL-6, type I IFN signature) (81)	Tissue IHC/serum	Cancer-induced nerve injury (CINI) promotes ICI resistance; reversible by targeting IL-6/IFNAR	Validated in murine models; no human prospective data	Preclinical; exploratory
ctDNA (MRD) (84, 85)	Plasma NGS	Dynamic monitoring of response and early relapse prediction	Cost, availability, need for assay harmonization	Emerging; prospective trials ongoing
Machine learning signatures (86–88)	Multi-omics	Integrates complex data for potentially superior predictive power	Overfitting risk, lack of external validation	Exploratory; no regulatory approval

CPS, combined positive score; IHC, immunohistochemistry; ICI, immune checkpoint inhibitor; MSI, microsatellite instability; dMMR, deficient mismatch repair; PCR, polymerase chain reaction; EBV, Epstein-Barr virus; ISH, *in situ* hybridization; TMB, tumor mutational burden; NGS, next-generation sequencing; PTEN, phosphatase and tensin homolog; UBT, urea breath test; ANA, antinuclear antibody; ENA, extractable nuclear antigen; IIF, indirect immunofluorescence; CLIA, chemiluminescence immunoassay; NLR, neutrophil-to-lymphocyte ratio; PLR, platelet-to-lymphocyte ratio; CBC, complete blood count; Treg, regulatory T cell; MDSC, myeloid-derived suppressor cell; NK, natural killer; SCFA, short-chain fatty acid; TAM, tumor-associated macrophage; PLD3, phospholipase D3; TLR9, toll-like receptor 9; ATF3, activating transcription factor 3; IFN, interferon; IFNAR, interferon-α/β receptor; ctDNA, circulating tumor DNA; MRD, minimal residual disease.

## 3 Autoantibodies: redefining the host–tumor interface

After reviewing the established canonical biomarkers that currently guide clinical decision making, we now turn to emerging host-centric candidates. Among these, autoantibodies have generated considerable interest as potential predictors of immunotherapy response, though their findings must be interpreted with caution and considered alongside validated biomarkers.

### 3.1 Definition and clinical context

Antinuclear antibodies (ANAs) target various components of the cell nucleus, including DNA, histones, and nuclear proteins, while antibodies against extractable nuclear antigens (ENAs) target specific ribonucleoprotein complexes. Both are classical serological markers of systemic autoimmune diseases but have recently garnered attention in oncology as potential indicators of pre-existing host immune activation that may influence response to immunotherapy (9, 45–48).

### 3.2 Autoantibodies as prognostic markers in gastric cancer

Zheng et al. conducted a retrospective study of 230 gastric cancer patients receiving immunotherapy (anti-PD-1/PD-L1 antibodies combined with chemotherapy) (8). The study reported that ENA positivity was significantly associated with longer OS (P = 0.046), while ANA positivity, ENA positivity, or combined ANA/ENA positivity was associated with longer PFS (all P < 0.05). In multivariate analysis, combined ANA/ENA status remained an independent predictor of PFS (HR 0.538, P < 0.001). The study did not investigate immune-related adverse events (irAEs); its findings are limited to the prognostic value of pre-existing autoantibodies.

### 3.3 Autoantibodies and immune-related adverse events

The relationship between autoantibodies and irAEs has been explored in other contexts. Elevated levels of pre-existing autoantibodies are proposed as one of the key mechanisms underlying irAE development during ICI therapy. Autoantibodies may contribute to irAEs through several pathways: (1) excessive activation of autoreactive T cells; (2) overproduction of inflammatory cytokines; (3) shared antigens between tumors and normal tissues; and (4) amplification of pre-existing abnormal antibodies (49).

The mechanistic model posits that blocking CTLA-4 can induce activation of autoreactive T cells by depleting Tregs and impairing their function, which in turn stimulates B cells to increase autoantibody production. Similarly, blocking PD-1 can reactivate exhausted T cells, leading to overactivation of autoreactive T cells. Epitope spreading can further break self-tolerance and trigger autoantibody-mediated tissue damage.

In the context of cancer, the incidence of grade ≥3 irAEs can be as high as 57.4% in patients receiving ICI combinations, with common irAEs including colitis, adrenal insufficiency, and thyroid dysfunction (49). While irAEs are often manageable with corticosteroids or immunosuppressants, severe irAEs may necessitate treatment interruption or discontinuation, underscoring the importance of identifying predictive biomarkers.

### 3.4 Methodological limitations and clinical translation

Current evidence linking autoantibody profiles to irAE prediction in gastric cancer remains limited. Most studies are retrospective, involve small sample sizes, and lack prospective validation. The exact mechanisms remain incompletely understood, and further large-scale prospective studies are required.

### 3.5 Mechanistic hypotheses

Several non-mutually exclusive hypotheses could explain the association between autoantibody induction and clinical benefit or toxicity (49):

- Baseline immune activation: Autoantibody positivity may be a surrogate for a more active, immune-fit host capable of mounting a robust response to immunotherapy (50).
- Epitope spreading: Tumor cell death induced by chemotherapy, vaccines, or ICIs could release nuclear antigens, amplifying a pre-existing autoimmune response and driving epitope spreading, thereby reinforcing anti-tumor immunity while also increasing irAE risk (51).
- Autoimmunity–ICI toxicity overlap: The presence of autoantibodies could identify patients at higher risk for irAEs, which have paradoxically been linked to better clinical outcomes in some studies (49, 52, 53).

### 3.6 Immunological perspective

Autoantibody positivity could reflect a pre-existing state of enhanced antigen presentation and T cell priming, possibly mediated by tumor-infiltrating B cells and tertiary lymphoid structures (TLS), which have been associated with favorable ICI outcomes in several solid tumors (49, 54, 55). In gastric cancer, Mori et al. demonstrated that patients with high TLS density showed excellent anti-tumor immune responses and a higher frequency of irAEs, suggesting that stronger immune activation underlies both efficacy and toxicity (50). The production of autoantibodies against tumor-associated antigens is thought to result from mutations, post-translational modifications, or aberrant degradation of self-proteins during tumorigenesis (45, 46, 56).

### 3.7 Context-dependent evidence

The evidence remains context-dependent. Toi et al. demonstrated that in non-small cell lung cancer patients treated with anti-PD-1 therapy, pre-existing autoantibodies were associated with longer PFS (52). However, other studies have linked ANA positivity to poorer outcomes in leukemia and ovarian cancer (57, 58). The International Consensus on ANA Patterns (ICAP) recognizes distinct staining patterns (e.g., homogeneous, speckled, nucleolar) with different clinical associations (59, 60). Whether specific patterns correlate with ICI response or irAE risk in GC remains unexplored.

### 3.8 Cross cancer evidence and clinical feasibility

Beyond ANA and ENA, autoantibodies against specific tumor-associated antigens (Tumor Protein p53 [p53], Mucin 1 [MUC1], etc.) have been studied in colorectal, lung, breast, and prostate cancers, suggesting their potential as diagnostic and prognostic tools (61–80). ANA testing is widely available, inexpensive, and non-invasive, making it an attractive candidate for integration into routine clinical workflows, particularly in resource-limited settings. However, its clinical utility as a predictive biomarker for ICI efficacy or irAE risk requires prospective validation.

## 4 Insights from murine models: translating mechanistic findings to human biomarkers

Preclinical murine models have provided critical mechanistic insights that can inform human biomarker development. The following subsections highlight key discoveries and their translational potential.

### 4.1 Cancer-induced nerve injury as a novel resistance mechanism

Baruch et al. (2025) demonstrated that cancer-induced nerve injury (CINI) promotes resistance to anti-PD-1 therapy in murine models of cutaneous squamous cell carcinoma, melanoma, and gastric cancer (81). Mechanistically, cancer cells degrade myelin sheaths of tumor-associated nerves; injured neurons initiate IL-6 and type I interferon-mediated inflammation to promote nerve healing; and chronic CINI skews the TME toward an immunosuppressive, exhausted state. Anti-PD-1 resistance in CINI models was reversible by denervation, conditional knockout of Activating Transcription Factor 3 (Atf3) in neurons, knockout of Ifnar1, or combining anti-PD-1 with anti-IL6 receptor blockade. These findings suggest that neuronal injury markers (ATF3, Proto-oncogene c-Jun [JUN]), neuroimmune crosstalk mediators (IL-6, type I interferons), and neurotropic bacterial signatures could serve as novel biomarkers in human GC (81).

### 4.2 The cholesterol-TFEB-PLD3-TLR9 axis in macrophage polarization

Tan et al. (2026) established a murine model of esophageal squamous cell carcinoma, revealing a cholesterol-TFEB-PLD3-TLR9 axis driving immunosuppressive tumor-associated macrophage polarization (82). Tumor secreted cholesterol induced TFEB nuclear translocation, which bound the PLD3 promoter to upregulate its expression. PLD3 localized to lysosomes and degraded single-stranded nucleic acids, suppressing TLR9 activation and impairing CD8+ T cell function. Therapeutic intervention with ODN2216-siPLD3 in murine models enhanced CD8+ T cell infiltration and significantly inhibited tumor growth. These findings identified PLD3 high macrophages as a promising diagnostic biomarker and therapeutic target (82).

### 4.3 Oncolytic virotherapy and chemo-resistant bystander CD8+ T cells

Yang et al. (2026) demonstrated that oncolytic virotherapy potentiates chemo-PD-1-immunotherapy by engaging chemo-resistant bystander CD8+ T cells (83). Within the TME, tumor-specific CD8+ T cells (TTST) are highly susceptible to chemotherapy-induced apoptosis, whereas virus-specific memory CD8+ T cells (TBYS) constitute a quiescent, chemotherapy-resistant population. An engineered oncolytic virus encoding TBYS cell epitopes (OV-BYTE) redirected these chemoresistant cells for tumor killing. This dual combination restored TTST cell function via reduced apoptotic susceptibility and acquisition of a polyfunctional, effector-like state. The translational potential was validated in patient-derived colorectal cancer organoids co-cultured with autologous PBMCs. These findings support the use of pre-existing antiviral immunity as a predictive biomarker and OV-BYTE as a rational combination strategy to overcome chemotherapy-induced T cell damage (83).

### 4.4 Translational biomarker concepts from murine models

These murine studies highlight several translatable biomarker concepts: (1) neuronal injury signatures (ATF3, IL 6, type I IFN signature); (2) macrophage polarization markers (PLD3, TFEB nuclear localization, TLR9 activity); (3) bystander T cell metrics (frequency of virus specific memory T cells, their chemo resistance profile); and (4) metabolic reprogramming markers (cholesterol uptake pathways, lysosomal function). Prospective validation in human GC cohorts is urgently needed to determine which of these murine-derived biomarkers translates to clinical utility.

## 5 Dynamic biomarkers and computational oncology

To overcome the limitations of static, single-analyte biomarkers, the field is moving toward more sophisticated, dynamic approaches.

### 5.1 Circulating tumor DNA

ctDNA is a rapidly evolving technology with transformative potential. As a liquid biopsy, it can capture tumor heterogeneity and clonal evolution in real time. In the perioperative setting, detection of ctDNA (MRD) after curative intent surgery is a powerful predictor of imminent recurrence (84). This opens the door for MRD-guided interventional trials, where treatment is escalated for ctDNA-positive patients and de-escalated for those who are negative. However, standardized thresholds, assay harmonization, and prospective interventional validation remain necessary before ctDNA-guided immunotherapy adaptation becomes routine practice (85). To date, ctDNA-guided treatment strategies remain investigational and are not yet part of standard clinical practice.

### 5.2 Machine learning (ML) and multi-omics

The complexity of tumor–immune interactions and the limited predictive capacity of conventional biomarkers have driven growing interest in ML-based approaches integrating transcriptomic and multi-omics data. A recent systematic review in gastrointestinal cancers reported that ML-derived gene signatures can achieve strong discriminatory performance for immunotherapy response, in some cases surpassing traditional biomarkers (86). For example, Wang et al. developed an antigen processing and presentation signature (APscore) that effectively predicted response to ICIs in advanced GC (AUC 0.85), and demonstrated that patients with a high APscore had significantly longer PFS (87). However, substantial heterogeneity in modeling strategies, frequent reliance on internal validation, and limited external prospective validation raise concerns about generalizability and overfitting. Larger, biomarker-stratified prospective studies are required before routine clinical implementation.

High-throughput technologies such as phage display, serological proteome analysis (SERPA), and protein microarrays have enabled simultaneous screening of thousands of autoantibodies, facilitating the discovery of multi-marker panels with improved diagnostic accuracy (75–80). External validation in large, prospective cohorts is essential before these models can be safely implemented in routine clinical care.

Importantly, except for MSI/dMMR and, to a lesser extent, PD-L1 CPS, most biomarkers discussed herein (TMB in GC, autoantibody profiling, inflammatory indices, ctDNA-guided adaptation, machine learning signatures) remain in various stages of clinical validation. Many derive from retrospective analyses, exploratory subgroup studies, or translational research cohorts. Prospective biomarker-driven trials with predefined stratification strategies are required before these tools can be incorporated into routine decision-making. Premature clinical implementation risks both overtreatment and inappropriate therapeutic exclusion.

## 6 Toward an integrated, host–tumor biomarker framework

Synthesizing this vast and sometimes conflicting body of evidence into a practical clinical approach requires conceptual structure. A hierarchical framework distinguishing validated predictive biomarkers from exploratory or hypothesis-generating markers may help prevent premature clinical translation while encouraging innovation. Within this hierarchy, MSI/dMMR and, with important limitations, PD-L1 CPS, represent the most clinically established predictive biomarkers in gastric cancer. In contrast, biomarkers such as autoantibody profiling, inflammatory indices, ctDNA-guided adaptation, TMB in GC, PTEN mutations, *H. pylori* status, and machine learning signatures remain investigational and require prospective, biomarker-stratified validation before routine adoption.

Historically, biomarker development in GC has been predominantly tumor-centric, focusing on tumor intrinsic genomic and immunologic features. The findings by Zheng et al. suggest that host-centric biomarkers may represent an underexplored but biologically meaningful complementary axis of prediction (8). This aligns with a broader paradigm shift in cancer biomarker research, where the integration of host immune responses, including autoantibody signatures, is increasingly recognized as essential for capturing the full complexity of tumor–host interactions (88–90).

**Figure 1:**
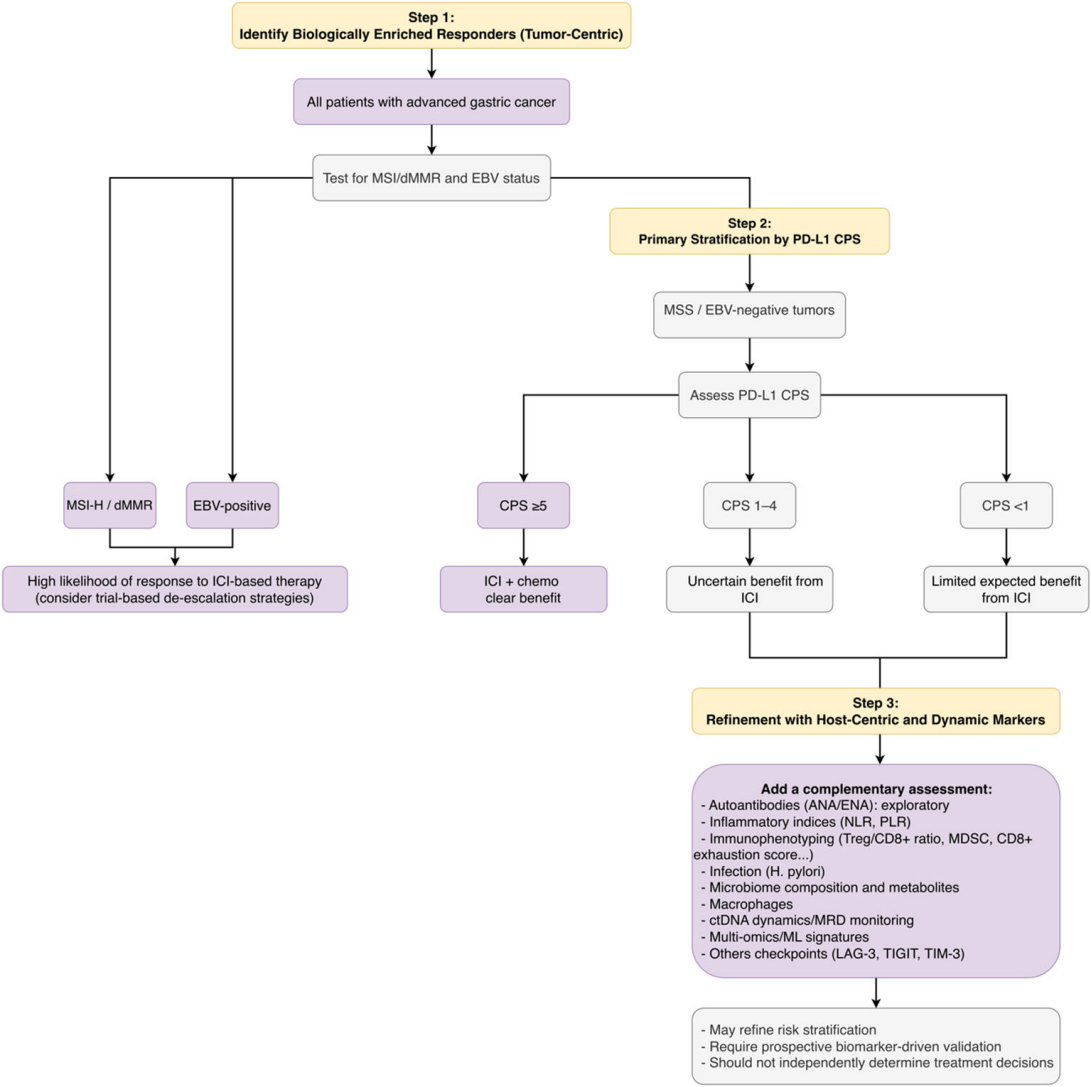
Proposed conceptual framework integrating tumor-centric and host-centric biomarkers in gastric cancer immunotherapy. This hypothesis-generating model illustrates a hierarchical integration strategy designed to refine patient selection for immune checkpoint inhibitor (ICI)-based therapy and is intended to stimulate prospective validation; it should not be used to guide clinical decisions outside of clinical trial contexts. Step 1 identifies biologically enriched responders through tumor-centric testing for MSI/dMMR and EBV status, with MSI-H/dMMR or EBV-positive patients constituting a small but highly responsive subgroup in whom de-escalation strategies may be considered within trials. Step 2 uses PD-L1 combined positive score (CPS) as the primary stratification tool for the majority of MSS/EBV-negative patients, with CPS ≥ 5 indicating clear benefit from ICI plus chemotherapy, CPS 1–4 indicating uncertain benefit, and CPS < 1 indicating limited expected benefit. Step 3 proposes refinement with host-centric and dynamic markers for the uncertain benefit group (CPS 1-4). These markers remain investigational and include: serum autoantibodies (ANA/ENA); systemic inflammatory indices (NLR, PLR); Treg/effector CD8+ T cell ratio, frequency of myeloid-derived suppressor cells (MDSC), CD8+ T cell exhaustion score (e.g., PD-1+TIM-3+), and NK cell degranulation/activating receptors; tumor genomics (PTEN mutations, TMB); H. pylori status; gut microbiome composition (e.g., Akkermansia muciniphila, SCFA producers) and microbial metabolites (succinate, butyrate, indole derivatives); PLD3-high tumor-associated macrophage signature; neuronal injury markers (ATF3, IL-6, type I interferon signature); ctDNA monitoring; and machine learning signatures. In addition, emerging immune checkpoints beyond PD-1 (LAG-3, TIGIT, TIM-3) and the controversial role of anti-CTLA-4 antibodies in Treg depletion versus CTLA-4 blockade on effector T cells are under active investigation as potential predictive biomarkers. All markers in Step 3 require prospective validation before routine implementation. The framework ultimately aims to transition from static, single-analyte biomarkers toward integrative, dynamic, systems-level models of patient selection.

We therefore propose a conceptual framework that integrates both domains (**Figure 1**). This framework is hypothesis-generating and should not guide clinical decisions outside clinical trials.

Step 1: Identify biomarker-enriched responders (tumor-centric); All patients should undergo testing for MSI/dMMR and EBV status. For these small but clinically significant subgroups, ICI-based therapy (with or without chemotherapy) is highly effective, and de-escalation in clinical trials is warranted.
Step 2: PD-L1 as a primary stratification tool (tumor-centric); For the majority of patients who are MSS and EBV-negative, PD-L1 CPS remains the primary stratification tool. Based on phase III trial data, patients with CPS ≥ 5 derive the clearest benefit from adding an ICI to chemotherapy.
Step 3: Refine with host-centric and dynamic markers; For patients with CPS 1–5 or <1, where benefit is less certain, additional markers can guide discussion:
  - Host serology: Pre-treatment autoantibodies (ANA/ENA), as suggested by Zheng et al., could identify a subset of patients with an “immune active” phenotype who might still benefit despite low PD-L1 expression. This hypothesis requires prospective validation within biomarker-stratified trials.
  - Systemic inflammation: Baseline NLR and other inflammatory indices can provide a rapid, inexpensive readout of the systemic inflammatory state.
  - Tumor genomics: TMB assessment may help identify patients with MSS tumors who have a higher likelihood of response, though the optimal threshold remains undefined. PTEN mutation testing, particularly in MSI-H tumors, may identify patients with intrinsic resistance who could benefit from combination strategies.
  - Microbiome status: H. pylori infection status may serve as a readily available, low-cost biomarker to further refine risk stratification.
  - Dynamic monitoring: Where available, ctDNA monitoring should be implemented to dynamically assess treatment response and detect molecular residual disease.

This framework remains conceptual. All proposed biomarkers beyond MSI/dMMR and PD-L1 CPS should be considered investigational and must undergo prospective validation before being incorporated into clinical decision-making. It is intended to stimulate prospective validation in biomarker-stratified studies, not to guide therapeutic decisions outside of clinical trial contexts. Ultimately, it underscores the need for a transition from single analyte biomarkers toward integrative, dynamic, and systems-level models of patient selection.

## 7 Future perspectives

Looking ahead, biomarker development for immunotherapy in GC must move beyond static, single-analyte testing. The complexity of both the tumor and the host immune system demands a more integrated, dynamic, and multidimensional approach.

### 7.1 The rise of integrative and dynamic biomarkers

The future lies in transitioning from single-point measurements toward continuous, dynamic assessment of the tumor–host interaction. Liquid biopsies, particularly ctDNA, are poised to play a central role. Serial ctDNA analysis has been shown to detect minimal residual disease (MRD) with high sensitivity and predict recurrence months before radiographic progression in gastroesophageal cancers (84, 85). Longitudinal ctDNA profiling can capture clonal evolution under the selective pressure of immunotherapy, identifying emerging resistance mutations in real time (91, 92), enabling adaptive therapeutic strategies.

Furthermore, the integration of diverse data streams, genomics, transcriptomics, proteomics, radiomics, and metabolomics, through machine learning and artificial intelligence, will be essential. ML-derived gene signatures have already outperformed traditional biomarkers such as PD-L1 in predicting ICI response (86, 93). The goal is to develop composite scores that incorporate not just TMB and PD-L1, but also host factors such as systemic inflammation (NLR, PLR), autoantibody profiles, CD8+ T cell exhaustion scores, NK cell degranulation capacity, Treg/effector CD8+ ratios, MDSC frequencies, ILC1/ILC3 ratios, and gut microbiome composition (including specific taxa and metabolites like succinate, butyrate, and indole derivatives).

### 7.2 Dissecting resistance mechanisms for rational combinations

A deeper understanding of resistance pathways is critical for improving outcomes. The identification of PTEN loss-of-function mutations as a mediator of intrinsic resistance in MSI-H tumors, coupled with the associated immunosuppressive macrophage-rich microenvironment, provides a rationale for PI3K/AKT/mTOR targeted combinations (18, 94). Myeloid-directed therapies (e.g., Colony-Stimulating Factor 1 Receptor [CSF-1R] inhibitors) are also under investigation.

Beyond PTEN, cancer-induced nerve injury (CINI) has emerged from murine models as a novel resistance mechanism. Injured neurons initiate IL-6 and type I interferon signaling, skewing the TME toward immunosuppression. Targeting IL-6 or Interferon-alpha/beta receptor (IFNAR) reverses anti-PD1 resistance, suggesting that neuronal injury markers (ATF3, JUN, IL-6, type I IFN signature) could serve as both predictive biomarkers and therapeutic targets (81). Similarly, the cholesterol Transcription Factor EB (TFEB) Phospholipase D family member 3 (PLD3) Toll-like receptor 9 (TLR9) axis identified in preclinical models points to PLD3 high macrophages as a potential biomarker and target for restoring TLR9-mediated immunity (82).

The detrimental impact of *H. pylori* infection on ICI efficacy, and the immunomodulatory role of specific gut microbial metabolites (succinate, butyrate), open avenues for microbiome modulation, including *H. pylori* eradication, probiotic supplementation (e.g., *Bifidobacterium*, *Lactobacillus rhamnosus GG*), and FMT, as low-cost adjuvants to enhance immunotherapy outcomes (39–44, 92, 95, 96).

### 7.3 The emergence of host-centric immune-oncology

Beyond autoantibodies, which signal a paradigm shift toward host-centric views, other immune components are gaining traction. LAG-3, TIGIT, and TIM-3 are inhibitory receptors expressed on exhausted CD8+ T cells, Tregs, and NK cells. Their co-expression patterns with PD-1 may identify patients who require combination checkpoint blockade. Ongoing clinical trials are evaluating dual blockade of PD-1 with LAG-3 or TIGIT. The spatial distribution of these molecules within the TME could refine biomarker strategies (20–22). High-throughput technologies such as phage display and protein microarrays now enable simultaneous screening of thousands of autoantibodies, facilitating the discovery of multi-marker panels with improved predictive accuracy. Recent studies have validated autoantibody signatures as independent predictors of ICI response in multiple cancer types, supporting their potential integration into clinical decision making (97–99).

The controversy surrounding anti-CTLA4 antibodies, whether they primarily deplete intratumoral Tregs via Fc-dependent ADCC or block CTLA 4 on effector T cells, has direct implications for biomarker development. Fc-engineered antibodies with enhanced ADCC (e.g., HCAb 4003-2) show superior Treg depletion and efficacy in preclinical models (32–38). Genotyping for FCGR3A (CD16) polymorphisms may help select patients likely to benefit from such agents.

Future research must focus on: (1) prospective validation of multi marker panels (autoantibodies, T cell exhaustion scores, NK function, neuronal injury markers, PLD3, microbiome metabolites) in large, biomarker stratified trials; (2) standardization of flow cytometry based immunophenotyping and assay platforms; (3) integrating host serology with tumor genomics to create a comprehensive “immune architecture” map for each patient; and (4) translation of murine derived biomarkers (CINI, PLD3) into human GC cohorts (100).

### 7.4 The imperative for prospective validation

A common thread across all emerging biomarkers, from autoantibodies to ctDNA dynamics to ML signatures, is the urgent need for rigorous prospective validation. The current evidence base for most novel markers is largely retrospective, exploratory, or derived from subgroup analyses, which are prone to bias and overfitting (102). Major collaborative efforts, such as AACR Project GENIE and the FDA-led MAQC, are now establishing frameworks for the clinical validation of multi-omic biomarkers (103). The next wave of progress will come from well-designed, prospective clinical trials that incorporate biomarker stratification as an integral part of their design, with clear pre-specified thresholds and decision algorithms.

In conclusion, the future of precision immunotherapy in gastric cancer is not about finding a single perfect biomarker, but in constructing interoperable biomarker ecosystems that integrate tumor genomics, host immunity (autoantibodies, immune cell functional states, microbiome), and real-time disease kinetics. This shift from a static, tumor-centric to a dynamic, host–tumor integrated paradigm is essential to realize the full promise of precision immune-oncology for every patient (104).

## 8 Conclusion

Emerging data on autoantibody profiling represent a valuable contribution to the biomarker field, reminding us that the host’s immune system holds important clues for predicting ICI response in GC. However, they are only one facet of a much broader host centric landscape that includes the gut microbiome and its metabolites (succinate, butyrate, indole derivatives), systemic inflammatory ratios (NLR, PLR, Treg/effector CD8+), CD8+ T cell exhaustion scores, NK cell functionality, and even neuronal injury signatures derived from murine models. All these host derived candidates require cautious interpretation and prospective validation before clinical translation.

At the same time, the field must not lose sight of established and emerging tumor intrinsic biomarkers. The molecular heterogeneity of GC, encompassing MSI, EBV, CIN, and GS subtypes, provides a critical framework for understanding differential ICI responsiveness. Within MSI-H tumors, further stratification by TMB and PTEN mutation status is essential to identify patients most likely to benefit from PD 1 monotherapy versus those who may require combination strategies. The identification of PTEN mutations as a negative predictor, and their association with an immunosuppressive TAM rich microenvironment, opens the door for rational combination therapies targeting the PI3K/AKT/mTOR axis or myeloid derived suppressor cells. Beyond PD 1, other immune checkpoints (LAG 3, TIGIT, TIM-3) are under active investigation as both biomarkers and therapeutic targets.

The next evolution of immunotherapy in gastric cancer will not be defined by discovering another single biomarker, but by constructing interoperable biomarker ecosystems that integrate tumor genomics, host immunity (including autoantibodies, microbiome, and immune cell functional states), and real-time disease kinetics. This paradigm shift must be grounded in rigorous prospective validation. At present, except for MSI/dMMR and PD L1 CPS, most biomarkers discussed remain investigational and should not guide routine clinical decisions. Moving from a tumor-centric to a host–tumor integrated paradigm is essential for realizing the full promise of precision immune-oncology.

## Glossary

AACR: American Association for Cancer Research
ADCC: antibody dependent cellular cytotoxicity
ANA: antinuclear antibody
ATF3: activating transcription factor 3
AUC: area under the curve
CBC: complete blood count
CD: cluster of differentiation
CIN: chromosomal instability
CLIA: chemiluminescence immunoassay
CPS: combined positive score
CSF1R: colony stimulating factor 1 receptor
ctDNA: circulating tumor DNA
dMMR: deficient mismatch repair
EBV: Epstein Barr virus
EFS: event free survival
ENA: extractable nuclear antigen
FCGR3A: Fc gamma receptor IIIa (CD16)
FMT: fecal microbiota transplantation
FOXP3: forkhead box P3
GC: gastric cancer
GENIE: Genomics Evidence Neoplasia Information Exchange
GPR109A: G protein coupled receptor 109A
GS: genomically stable
GSDME: gasdermin E
HLA DR15: human leukocyte antigen DR15
HR: hazard ratio
ICAP: International Consensus on ANA Patterns
ICI: immune checkpoint inhibitor
IFN: interferon
IFNAR: interferon α/β receptor
IHC: immunohistochemistry
IIF: indirect immunofluorescence
ILC: innate lymphoid cell
irAE: immune related adverse event
ISH: *in situ* hybridization
JUN: proto oncogene c Jun
LAG3: lymphocyte activation gene 3
LDH: lactate dehydrogenase
MAQC: Microarray Quality Control Consortium
MDSC: myeloid derived suppressor cell
mIHC/IF: multiplex immunohistochemistry/immunofluorescence
ML: machine learning
MRD: minimal residual disease
MSI: microsatellite instability
MSI-H: microsatellite instability high
MSS: microsatellite stable
mTOR: mammalian target of rapamycin
NF-κB: nuclear factor kappa light chain enhancer of activated B cells
NGS: next generation sequencing
NK: natural killer
NLR: neutrophil to lymphocyte ratio
ORR: objective response rate
OS: overall survival
OV-BYTE: oncolytic virus encoding bystander T cell epitopes
PBMC: peripheral blood mononuclear cell
PCR: polymerase chain reaction
PD-1: programmed cell death protein 1
PD-L1: programmed death ligand 1
PFS: progression free survival
PLD3: phospholipase D3
PLR: platelet to lymphocyte ratio
PTEN: phosphatase and tensin homolog
SCFA: short chain fatty acid
SERPA: serological proteome analysis
